# Complete Genome Sequences of Four Atrazine-Degrading Bacterial Strains, *Pseudomonas* sp. Strain ADPe, *Arthrobacter* sp. Strain TES, *Variovorax* sp. Strain 38R, and *Chelatobacter* sp. Strain SR38

**DOI:** 10.1128/MRA.01080-20

**Published:** 2021-01-07

**Authors:** Loren Billet, Marion Devers-Lamrani, Rémy-Félix Serre, Emmanuel Julia, Céline Vandecasteele, Nadine Rouard, Fabrice Martin-Laurent, Aymé Spor

**Affiliations:** a AgroSup Dijon, INRAE, Université Bourgogne Franche-Comté, Agroécologie, Dijon, France; b INRAE, US 1426, GeT-PlaGe, GenoToul, Castanet-Tolosan, France; University of Arizona

## Abstract

We report here the complete genome sequences of four atrazine-degrading bacteria. Their genomes will serve as references for determining the genetic changes that have occurred during an evolution experiment.

## ANNOUNCEMENT

We recently set up an evolution experiment on a four-species bacterial consortium to determine whether liquid medium supplemented with the herbicide atrazine and/or different N and C sources could support the coexistence of multiple species (1). Here, we provide the full genome sequences of the four ancestral atrazine-degrading strains, namely, *Pseudomonas* sp. strain ADPe (2, 3), *Variovorax* sp. strain 38R (4), *Arthrobacter* sp. strain TES (5), and *Chelatobacter* sp. strain SR38 (6).

The strains were all derived from ancestors isolated from atrazine-contaminated soils and were stored at −80°C in glycerol (30%). Multiple colonies were picked and grown to late exponential phase on mineral salt medium containing atrazine as the sole nitrogen source. Genomic DNA was extracted using the Qiagen genomic DNA extraction kit. Oxford Nanopore Technologies (ONT) libraries were generated from 2 µg fragmented DNA with a ligation sequencing kit (SQK-LSK109) and sequenced on the GridION platform using an R9.5 flow cell. Illumina paired-end (PE) reads were prepared using the TruSeq Nano DNA LT library preparation kit (Illumina). Briefly, DNA was fragmented by sonication and adaptors were ligated. Eight cycles of PCR were applied to amplify the libraries. Library quality was assessed using Fragment Analyzer (Advanced Analytical Technologies, Inc.), and libraries were quantified by quantitative PCR using the Kapa library quantification kit (Roche). Sequencing was performed on a HiSeq instrument (Illumina) using a PE read length of 2 × 150 bp with the Illumina HiSeq 3000 reagent kits.

Fast5 files from ONT sequencing were obtained with ONT MinKNOW software (v1.10.24-1) and were base called with ONT Albacore sequencing pipeline software (v2.1.10). Adaptors were trimmed using Porechop v0.2.1 (7), and reads with a quality score of <7 and size of <3,000 bp were discarded using NanoFilt v2.2.0 (8). Nanopore reads were then assembled using Canu v1.7 (9) with the “minReadLength=3000” and “genomeSize” options. A first polishing step and circularization were performed on the assembly using Pilon v1.22 (10) and Circlator v1.5.1 (11), respectively. Illumina PE reads were processed with Trim Galore v0.4.0 (https://github.com/FelixKrueger/TrimGalore) to trim adaptor sequences and were mapped on the assembly using BWA-MEM v0.7.12 (12) and SAMtools v1.3.1 (13). The mapping was finally used to improve the polishing with two rounds with Pilon v1.22 with the following option: mindepth, 25. For each strain, taxonomic classification was inferred using MiGA (14) and the maximum average amino acid identity (AAI) found between its chromosome and all of the reference genomes in the NCBI RefSeq database. Default parameters were used for all software unless otherwise noted. Detailed statistics regarding the assembly of the four genomes are given in Table 1.

**TABLE 1:** Assembly statistics and accession numbers for the strains in this study

Strain	No. of reads	Nanopore read coverage (×)	*N*_50_ (nucleotides)	No. of contigs	Contig	Contig size (bp)	GenBank accession no.	GC content (%)
*Pseudomonas* sp. ADPe	165,586	366	21,830	2	Chromosome	7,177,635	CP062122	66.9
					pADPe	62,583	CP062123	64.7
*Variovorax* sp. 38R	308,810	571	15,149	1	Chromosome	6,870,625	CP062121	67.5
*Arthrobacter* sp. TES	153,561	633	25,143	4	Chromosome	4,181,416	CP062235	63.5
					pTES1	351,150	CP062236	62
					pTES2	205,802	CP062237	62.6
					pTES3	48,156	CP062238	60.9
*Chelatobacter* sp. SR38	116,302	290	23,740	9	Chromosome	5,667,809	CP062112	63.4
					pSR1	560,213	CP062113	63.2
					pSR2	120,900	CP062114	61.2
					pSR3	170,880	CP062115	58.8
					pSR4	74,540	CP062116	58.0
					pSR5	163,970	CP062117	60.4
					pSR6	52,741	CP062118	58.3
					pSR7	359,029	CP062119	60.2
					pSR8	197,271	CP062120	62.8

Taxonomic classification of *Pseudomonas* sp. ADPe confirmed that it may belong to the species Pseudomonas citronellolis (*P* = 0.03) (15). The chromosome contains five 16S rRNAs. The sequence of its circular plasmid is similar to that of pADP1 (99% overall similarity) except that a 22.3-kb sequence containing *atzA* and *atzB* and surrounded by insertion sequences (ISs) is deleted.

*Variovorax* sp. 38R most likely belongs to the genus *Variovorax* (*P* = 0.0049) and probably belongs to the species Variovorax paradoxus (*P* = 0.083). It contains *atzA* and *atzB* in two distinct regions, both delimited by ISs and exhibiting 99% similarity to *Pseudomonas* sp. strain ADP1.

*Arthrobacter* sp. TES most likely belongs to the same species as *Arthrobacter* sp. strain ZXY-2 (*P* = 0.0054). Atrazine-degrading genes *trzN*, *atzB*, and *atzC* are all located on the 205.8-kbp contig; *trzN* is found within a 9,081-bp region and *atzB* and *atzC* within a 36,366-bp region, both of which present 99% similarity to the atrazine-degrading plasmid pTC1 of Paenarthrobacter aurescens strain TC1 (GenBank accession number CP000475.1).

For *Chelatobacter* sp. SR38, AAI analysis performed on its chromosome reveals that it belongs to the genus *Aminobacter* and probably to the species Aminobacter aminovorans (*P* = 0.035). A BLASTN analysis indicated that *atzA* is located on the 74-kb uncircularized contig while *atzB*, *atzC*, and *trzD* are on the 197-kb one. Interestingly, *atzB* is present in two copies on this contig.

These genomes will be used as references to analyze full genome resequencing of these strains to search for genetic changes that might have occurred in the time course of their evolution in a four-species atrazine-degrading bacterial consortium facing different environmental challenges.

### Data availability.

Raw data and assembled genomes have been deposited in the NCBI GenBank under the BioProject accession number PRJNA664737. GenBank accession numbers for the assembled genomes are given in Table 1.
